# Entropic barrier of water permeation through single-file channels

**DOI:** 10.1038/s42004-023-00919-0

**Published:** 2023-06-29

**Authors:** Johann Wachlmayr, Gotthold Fläschner, Kristyna Pluhackova, Walter Sandtner, Christine Siligan, Andreas Horner

**Affiliations:** 1 Institute of Biophysics, Johannes Kepler University Linz, Linz, Austria; 2 Department of Biosystems Science and Engineering, Eidgenössiche Technische Hochschule (ETH) Zürich, Basel, Switzerland; 3 Stuttgart Center for Simulation Science, Cluster of Excellence EXC 2075, University of Stuttgart, Universitätsstr. 32, 70569 Stuttgart, Germany; 4 Center of Physiology and Pharmacology, Institute of Pharmacology, Medical University of Vienna, Schwarzspanierstr. 17A, 1090 Vienna, Austria

**Keywords:** Biophysical chemistry, Membrane proteins, Membrane proteins, Biophysical chemistry, Characterization and analytical techniques

## Abstract

Facilitated water permeation through narrow biological channels is fundamental for all forms of life. Despite its significance in health and disease as well as for biotechnological applications, the energetics of water permeation are still elusive. Gibbs free energy of activation is composed of an enthalpic and an entropic component. Whereas the enthalpic contribution is readily accessible via temperature dependent water permeability measurements, estimation of the entropic contribution requires information on the temperature dependence of the rate of water permeation. Here, we estimate, by means of accurate activation energy measurements of water permeation through Aquaporin-1 and by determining the accurate single channel permeability, the entropic barrier of water permeation through a narrow biological channel. Thereby the calculated value for $\triangle {S}^{{{\ddagger}} }$ = 2.01 ± 0.82 J/(mol·K) links the activation energy of 3.75 ± 0.16 kcal/mol with its efficient water conduction rate of ~10^10^ water molecules/second. This is a first step in understanding the energetic contributions in various biological and artificial channels exhibiting vastly different pore geometries.

## Abstract (web summary)

Facilitated water permeation through narrow biological channels is fundamental for all forms of life, but despite its significance the energetics of water permeation are still elusive. Here, the authors use accurate activation energy and single channel permeability measurements to estimate the entropic barrier of water permeation through Aquaporin-1.

## Introduction

Water transport across lipid membranes is fundamental for all forms of life. Thereby, passive water flux is facilitated by aquaporins^1,2^ as well as ion channels^3^ and transporters^4^ as was recently shown. Water permeation through such narrow biological channels occurs partly dehydrated. This is, bulk water loses two of its approximately four hydrogen bonds with neighboring water molecules subsequent to which it enters the single-file region of the water channel (e.g. aquaporin). These hydrogen bonds can be substituted with hydrogen bond acceptors and/or donors of channel lining amino acid residues^5^. Together with the dehydration at the channel entrance and temporary closings of the pore, this results in an activation energy (${E}_{a}$) for water permeation^6^. In a similar manner, water can permeate through ion channels^3^ and artificial water channels. The latter are developed to create bioinspired membranes for chemical separations and engineering applications^7^. These engineered channels are designed with the aim to reach permeability and selectivity properties matching those seen in biological systems.

Measurements of ${E}_{a}$ can be used to prove the presence of water pores without the need to employ channel inhibitors^8^. This approach is now commonly used for the characterization of artificial water channels^7,9–15^. *E*_*a*_s of biological water channels such as aquaporins (AQPs) are on the order of 4–5 kcal/mol^16^, similar to *E*_*a*_ for self-diffusion of water molecules in bulk ( ~ 4.6 kcal/mol)^17^. In contrast, unfacilitated water permeation through the lipid bilayer exhibit *E*_*a*_s > 10 kcal/mol^18^. *E*_*a*_*s* can be estimated from temperature dependent water permeability measurements utilizing Arrhenius plots. However, to obtain a better understanding of the nature of the investigated reaction knowledge of all energetic contributions is necessary. In this regard, the experimentally accessible ${E}_{a}$ is a good approximation for the enthalpic barrier ∆H^‡^^19^. The relationship between enthalpy and activation energy *E*_*a*_ for a constant pressure process is^20^

1 $$\triangle {H}^{{{\ddagger}} }={E}_{a}-{RT}+P(\triangle {V}^{{{\ddagger}} })$$

where *P* and ∆*V*^‡^ are the pressure and the volume of activation. For unimolecular reactions and to a very good approximation for reactions in solution ∆*V*^‡^ = 0 ^20^. The enthalpic barrier is related to the entropic barrier ∆*S*^‡^ and the Gibbs free energy of activation ∆*G*^‡^ via

2 $$\triangle {G}^{{{\ddagger}} }=\triangle {H}^{{{\ddagger}} }-T\triangle {S}^{{{\ddagger}} }$$

Recently, we showed by utilizing transition state theory that the single channel water permeability *p*_*f*_ and ∆G^‡^ are intricately linked^8,19^.

3 $${p}_{f}=\frac{{v}_{0}{V}_{W}}{{N}_{A}}{{\exp }}\left(-\frac{\triangle {{{{{{\rm{G}}}}}}}^{{{\ddagger}} }}{{RT}}\right)$$

where ${v}_{0}=\frac{{k}_{B}T}{h}$, *V*_*W*_, *N*_*A*_, *R*, *h*, *k*_*B*_ and T are the attempt frequency, the molar volume of water, Avogadro’s constant, the molar gas constant, Planck’s constant, Boltzmann constant and the absolute temperature, respectively. Thereby, high *p*_*f*_ values are linked to low values of ∆G^‡^. Interestingly, experimental values found for artificial channels seem not to be in agreement with Equation 3 by reporting high *E*_*a*_ and high *p*_*f*_ values^10–14^. This inconsistency may be explained threefold: Erroneous estimation of (i) *p*_*f*_ and/or (ii) *E*_*a*_ values. Whereas the former is already a matter of debate^6,16,21,22^, the latter is testified by significant variations in published *E*_*a*_ values^16^, e.g. values for AQP1, AQPZ, and narrow carbon nanotube porins (nCNTP) vary between 3.1 – 5.1 kcal/mol^2,9,13,23,24^, 2.1 – 6.1 kcal/mol^25–30^, and 5.3 – 24.1 kcal/mol^13,14^, respectively. Furthermore, (iii) an unknown entropic gain ∆S^‡^ (positive ∆S^‡^) could theoretically overwhelm the enthalpic contribution enabling the mathematical compatibility of high *p*_*f*_ and *E*_*a*_ values (see Equation 2). However, experimental evidence for the third possibility is missing.

To measure the entropic contribution on the permeation of water molecules through single-file channels, we overexpressed, purified and reconstituted AQP1 in large unilamellar vesicles. Optimized measurement conditions and an improved data analysis procedure for large unilamellar vesicles (subjected to a hyperosmotic gradient at different temperatures utilizing stopped-flow methodology) enabled us to calculate an accurate *E*_*a*_ for water permeation through AQP1. This in combination with a precise *p*_*f*_ from our previous work^5^ and the determination of the transmission coefficient *κ* by performing molecular dynamics simulations of water passage through AQP1 to obtain estimates for the probability of recrossing the energy barrier, allowed us to gauge the entropic component of the activation barrier for water transport through single-file channels. This analysis can be extended to other water channels to assess possible variabilities due to different channel architectures.

## Results

### Error sources and error propagation in estimating *E*_*a*_

#### Background correction

The *E*_*a*_ of water permeation is experimentally accessible via temperature dependent measurements of the rate of water flux *k*, or parameters proportional to *k* (e.g. *p*_*f*_ or more commonly *P*_*f*_, being the water permeability of the sample). The activation energy *E*_*a*_ can be obtained from an Arrhenius plot, assuming that *k* obeys the Arrhenius equation:

4 $$k=\bar{A}{e}^{-{E}_{a}/{RT}}$$

where $\bar{A}=\gamma A$ has a neglectable temperature dependence. γ is a constant which depends on the variable (*k*, *p*_*f*_, *P*_*f*_) used in the Arrhenius plot.

Drawing ${{{{{{\rm{ln}}}}}}}({P}_{f})$ vs ${T}^{-1}$ results in a straight line where the slope corresponds to the term $({-E}_{a}/R)$. The ${P}_{f}$ value of a membrane containing channels represents the sum of ${P}_{f,m}$, the permeability coefficient of the channel-free membrane, and ${P}_{f,c}$, the permeability coefficient of the channel, respectively^29^.

5 $${P}_{f}={P}_{f,m}+{P}_{f,c}={P}_{f,m}+\frac{n\cdot {p}_{f}}{\pi \cdot {d}^{2}}$$

Hence, for the determination of ${E}_{a}$ of the channel, ${P}_{f,c}$ or ${p}_{f}$ has to be used instead of ${P}_{f}$. This is typically achieved by subtraction of ${P}_{f,m}$ from the ${P}_{f}$ obtained for the channel-doped membrane. ${p}_{f}$ can be calculated from the resulting ${P}_{f,c}$ by its division with the number *n* of channels per vesicle and by multiplying it with the membrane area of the vesicle (with *d* being the diameter of the vesicle). This correction is, however, often neglected in the literature. The failure to account for water diffusion through the membrane may explain the scatter of published ${E}_{a}$ literature values^16^ of AQP1^2,9,13,24,31^ and AQPZ^25,26,28–30,32^ (see Supplementary Figure 1**)**. It is evident that the impact of a missing background subtraction is more pronounced if ${P}_{f,c}$ approaches ${P}_{f,m}$.

#### Inaccuracy of water permeability estimation

**Fig. 1:**
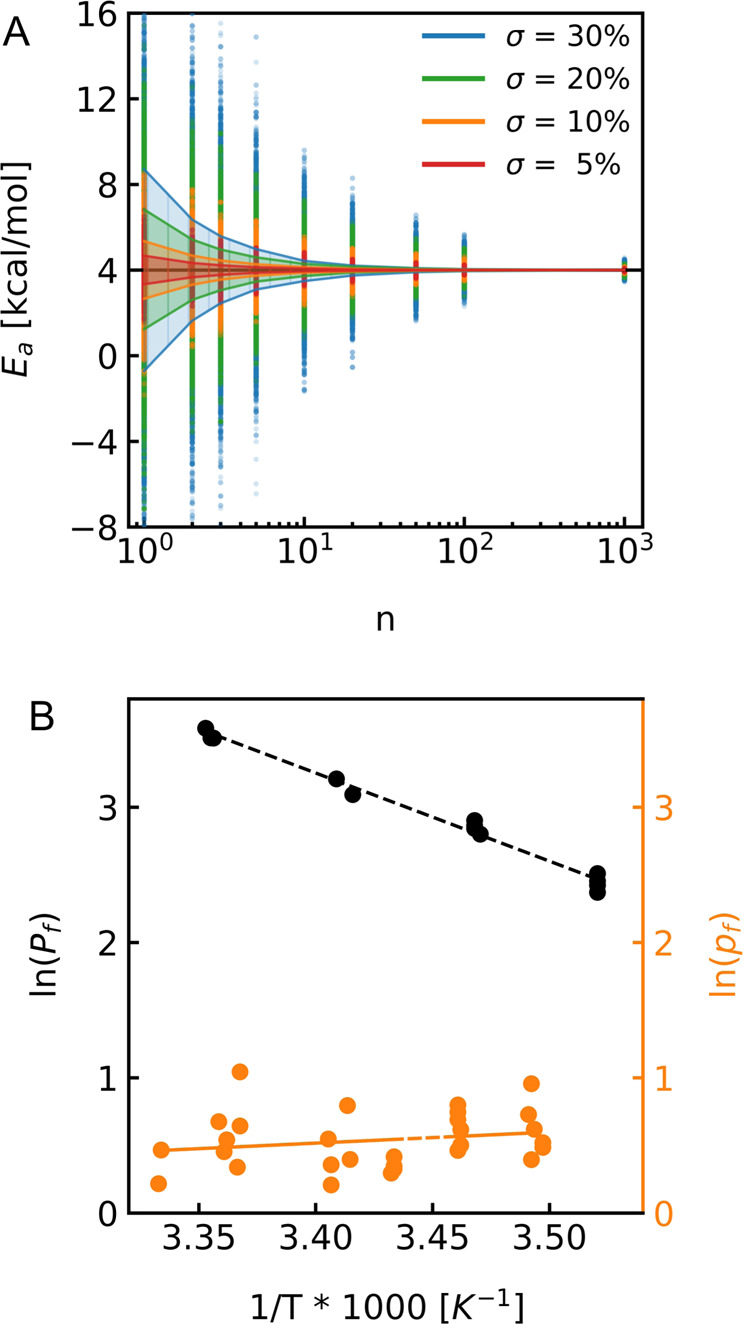
*E*_*a*_ depends on the accuracy of water permeability estimation. **A** Mean *E*_*a*_ values of n experiments calculated from Arrhenius plots (see Figure S3) using *P*_*f*_ values with a standard deviation ${{{{{{\rm{\sigma }}}}}}}_{{{{P}}}_{{{f}}}}$ of 30% (blue), 20% (green), 10% (orange) and 5% (red) represented as dots. The standard errors of the mean depending on the number of measurements n are shown as solid lines for the corresponding values of ${{{{{\rm{\sigma }}}}}}$. **B** Exemplary Arrhenius plots of polar lipid extract (PLE) (*E*_*a*_ = 11.6 kcal/mol) only (black dots) and AQP1 (*E*_*a*_ = −1.62 kcal/mol) containing (orange dots) GUVs. *P*_*f*_ was measured utilizing the micropipette aspiration technique equipped with a homebuilt temperature control system. To calculate p_f_ values the density of AQP1-YFP was counted with fluorescence correlation spectroscopy. Corresponding fits to the data are shown.

Temperature dependent water permeability measurements are most frequently conducted with large unilamellar vesicles utilizing the stopped-flow methodology. However, also scanning electrochemical microscopy on free standing planar lipid bilayers was employed successfully^29^. In addition, the recently published micropipette aspiration-based water permeability assessment with giant unilamellar vesicles (GUVs)^33^ can be adapted with a temperature control system to allow for ${E}_{a}$ measurements. These methods have in common that the accuracy of estimated ${E}_{a}$ values greatly depends on the accuracy of ${P}_{f}$ estimation. Figure 1A illustrates a simulation with varying accuracies (standard deviation σ) of Gaussian distributed *P*_*f*_ estimations for five temperatures (283 K, 287 K, 291 K, 295 K, 299 K). *E*_*a*_ was determined from the slope of the corresponding Arrhenius plot (Supplementary Figure 2**)**, according to Equation 4, and plotted in Fig. 1A. The simulation revealed that 20, 14, 7, 4 measurements for each temperature were necessary to reach a standard error of the mean of less than 5% for a ${\sigma }_{{P}_{f}}$ of 30%, 20%, 10%, and 5% respectively. Whereas, the micropipette aspiration technique is capable of estimating *E*_*a*_ of passive water permeability through the lipid bilayer with high fidelity (Fig. 1B) a σ of approx. 30% limits its applicability for *E*_*a*_ estimation through membrane channels. Thereby, the incompatibility of measuring the same GUV at different temperatures, necessitates *p*_*f*_ values instead of ${{P}_{f,c}}$ values to be used in the Arrhenius plots (Fig. 1B). However, in this case the additional uncertainty of channel counting increases σ by 20% compared to lipid only measurements. Hence, this approach would require at least 80 measurements to calculate *E*_*a*_ for AQP1 with satisfactory accuracy. For stopped-flow methodology the situation is different. The same vesicle suspension can be subjected to hyperosmotic buffer conditions at different temperatures. With a σ of ~5% for repetitive measurements of the same vesicle population and ~ 13% for independently prepared empty lipid vesicles (Supplementary Figure 2) this method is well suited to provide accurate estimates of *E*_*a*_ for membrane channels.

#### Implications of the temperature range

**Fig. 2:**
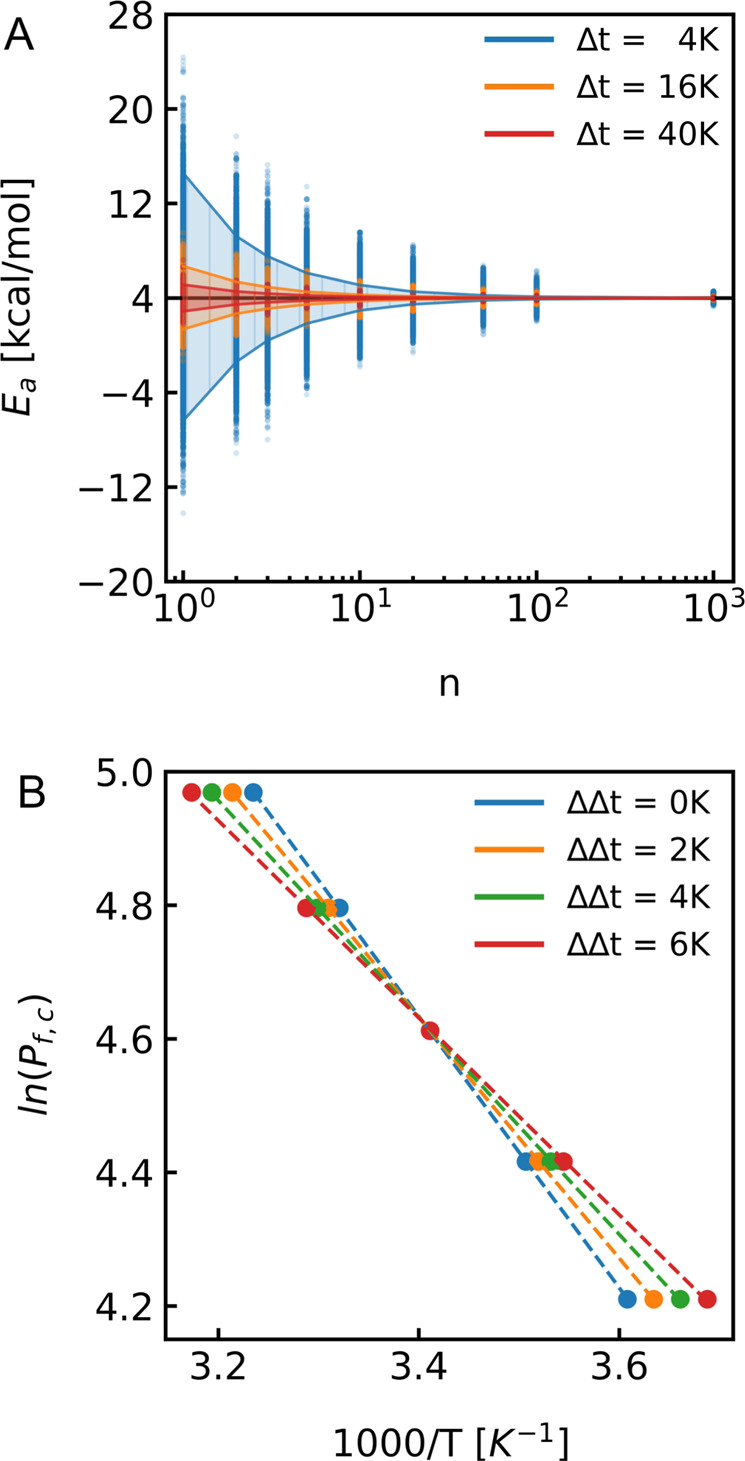
The experimental temperature range and its consequences. **A** Confidence interval of ${{{{{{\rm{E}}}}}}}_{{{{{{\rm{a}}}}}}}$ for different temperature ranges and a ${\sigma }_{{P}_{f}}$ of 10%. Values of ${E}_{a}$ which were calculated from an Arrhenius plot using ${P}_{f}$ values and temperature ranges of 4 K (288 K, 289 K, 290 K, 291 K, 292 K) (blue), 16 K (283 K, 287 K, 291 K, 295 K, 299 K) (orange) and 40 K (277 K, 287 K, 297 K, 307 K, 317 K) (red) are represented as dots. The solid lines show the corresponding confidence intervals for the mean *E*_*a*_s. Plots for a ${\sigma }_{{P}_{f}}$ of 20% and 30% can be found in Figure S5. **B** Effect of inaccurate temperature control: a linear error in temperature of 0 K (blue), 2 K (orange), 4 K (green) and 6 K (red) results in an error in ${E}_{a}$ of 1% (blue), 11% (orange), 20% (green) and 28% (red) with a difference less than 1% for globally fitted ${E}_{a}$ values from analytical fits.

Exemplary temperature ranges used in literature to estimate *E*_*a*_ via Arrhenius plots range from 281 to 286 K for *Hs*AQP2^24^ in yeast secretory vesicles or 283 to 298 K for pR-pillar5ene^10^ to 284 to 323 K for nCNTP^13^. To investigate the influence of the temperature range on *E*_*a*_ values we utilized our simulation approach with Gaussian distributed *P*_*f*_ estimates with varying accuracies. *P*_*f*_s were simulated for five temperatures within different temperature ranges (Supplementary Fig. 3). Fig. 2A and Supplementary Fig. 4 show that the temperature range, Δt, chosen for the Arrhenius plot greatly determines the accuracy of calculated *E*_*a*_ values. Thereby, the number of measurement points within this temperature interval has a marginal influence on *E*_*a*_ (Supplementary Fig. 4). It is important to note that the temperature set at the cooling device during measurements does not need to be the same as in the measurement cuvette. Depending on the insulation of the system such deviations ΔΔt in Δt can amount to up to several degrees Kelvin. Compared to assumed measurements conditions ranging from 277 K to 309 K, real conditions with a *ΔΔt* of 2 K, 4 K, and 6 K lead to an overestimation of *E*_*a*_ by 11%, 20%, and 28%, respectively (Fig. 2B).

#### Peculiarities of proteoliposomes

**Fig. 3:**
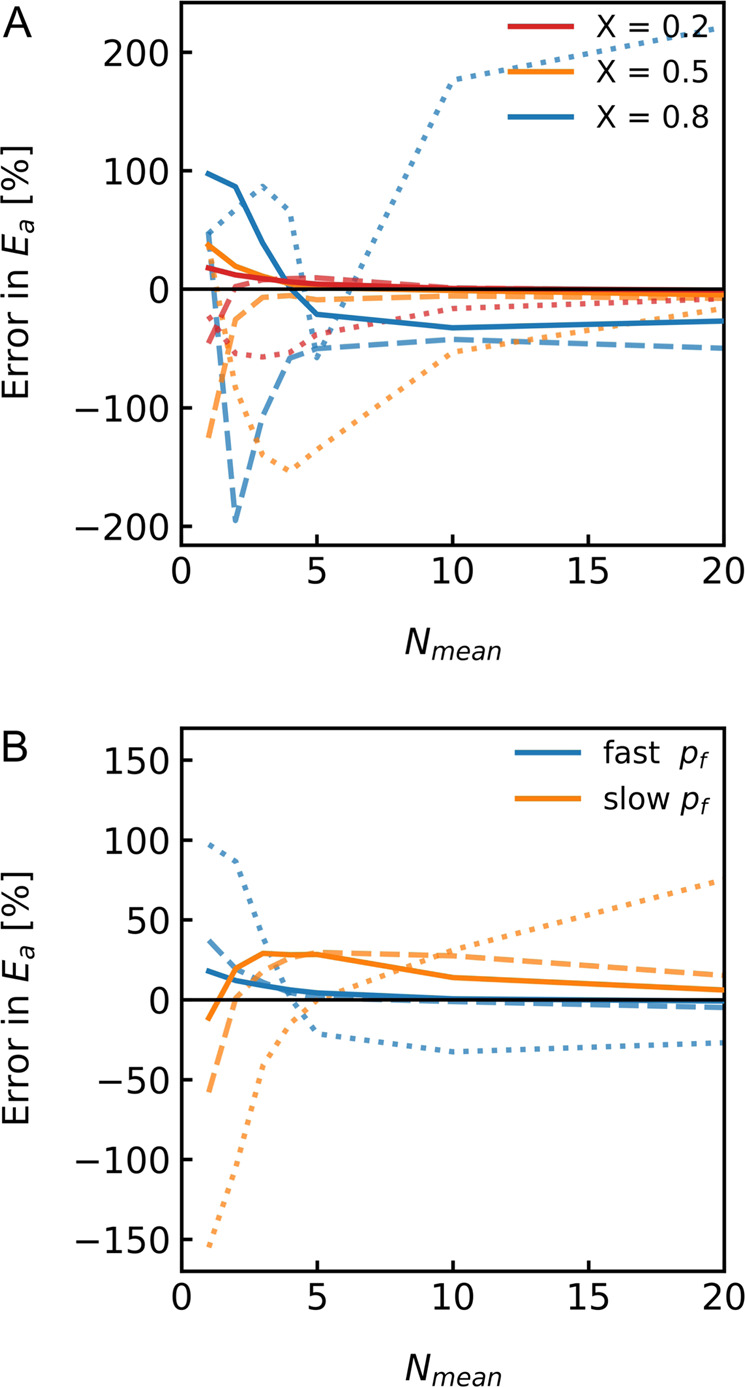
*E*_*a*_ depends on the reconstitution efficiency. **A** Error in ${{{{{{\rm{E}}}}}}}_{{{{{{\rm{a}}}}}}}$ is plotted over the average number of monomers per proteoliposome N_mean_ for an empty liposome fraction X of 20% (red), 50% (orange) and 80% (blue) fitted with an exponential function (dotted lines), the analytical solution (dashed lines) or the global ${{{{{{\rm{E}}}}}}}_{{{{{{\rm{a}}}}}}}$ fit (solid lines). **B** Error in ${{{{{{\rm{E}}}}}}}_{{{{{{\rm{a}}}}}}}$ depending on the relation ${{{{{{\rm{P}}}}}}}_{{{{{{\rm{f}}}}}},{{{{{\rm{m}}}}}}}$ (6 µm/s corresponding to large unilamellar vesicles (LUVs) composed of E. coli polar lipids (PLE)^22^) and ${{{{{{\rm{P}}}}}}}_{{{{{{\rm{f}}}}}},{{{{{\rm{c}}}}}}}$. Error in ${{{{{{\rm{E}}}}}}}_{{{{{{\rm{a}}}}}}}$ is plotted over the average number of monomers per proteoliposome N_mean_ for an empty liposome fraction X of 20% (solid lines), 50% (dashed lines) and 80% (dotted lines) for a fast ${{{{{{\rm{p}}}}}}}_{{{{{{\rm{f}}}}}}}$ of 3.2 · 10^−13^ cm^3^/s (blue) and a slow ${{{{{{\rm{p}}}}}}}_{{{{{{\rm{f}}}}}}}$ of 2.0 · 10^−14^ cm^3^/s (orange).

Membrane protein reconstitution into proteoliposomes (PLs) results in a fraction of empty vesicles. The fraction of liposomes containing membrane proteins may vary but is thought to exhibit a Poisson distribution of channel protomers per PL^5^. We recently showed that the varying reconstitution efficiency causes errors in estimating *P*_*f*_ due to fitting artefacts, which are larger at a smaller average number of protomers per PL, N_mean_. Moreover, the effect is more pronounced at smaller differences between ${P}_{f,m}$ and *P*_*f,c*_^22^. To illustrate its implications on *E*_*a*_ estimation we simulated a vesicle population consisting of an empty vesicle fraction and PLs with varying amounts of Poisson distributed channels. As can be seen in Fig. 3A the accuracy of *E*_*a*_ estimation increases with decreasing empty vesicles fraction X. Notably, the error in *E*_*a*_ depends on the fitting routine used to evaluate the stopped-flow raw data. The largest error was found using our recently established approximation based on exponential fits to the data (dotted lines in Fig. 3A)^21^. The error in *E*_*a*_ was significantly reduced using the analytical solution (dashed lines in Fig. 3A). However, we obtained the best results by far using a global analytical fit to a measurement series with one vesicle suspension subjected to hyperosmotic gradients at different temperatures. Since, the same vesicle suspension is used in stopped-flow experiments it is possible to fit the data with one global empty vesicle fraction *X* with a fixed *P*_*f,m*_ and more importantly one global value for *E*_*a*_. However, Fig. 3B highlights that the accuracy of *E*_*a*_ still depends on the actual water transport capacity of the channel under investigation. In other words, if the unitary water permeability *p*_*f*_ is high as in the case of AQP1, the error in *E*_*a*_ is smaller. If on the other hand *p*_*f*_ is smaller as compared to AQP1, this reduces the difference in ${P}_{f,m}$ and ${P}_{f,c}$ and increases the uncertainties in the fitting procedure.

### Activation energy of water permeation through AQP1

**Fig. 4:**
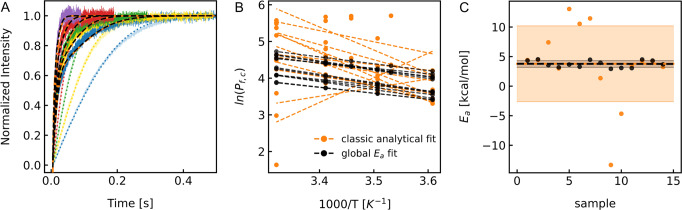
Temperature dependent water flux measurements of AQP1 proteoliposomes. **A** Representative osmotic water flux measurements at 277 K (blue), 283 K (yellow), 289 K (green), 295 K (red), and 301 K (purple). Vesicles (100 mM NaCl, 10 mM MOPS, pH 7.4) were rapidly mixed with hyperosmotic buffer (0.5 M sucrose, 100 mM NaCl, 10 mM MOPS, pH 7.4). Light colored curves represent measurements of liposomes without protein with the corresponding fits (dotted lines). Dark colored curves show AQP1 proteoliposome traces. Global *E*_*a*_ fits and classic analytical fits are monitored as dashed black and dash-dotted orange lines, respectively. Dashed black lines superpose dash-dotted orange lines. **B** Arrhenius plot, showing the natural logarithm of ${{{{{{\rm{P}}}}}}}_{{{{{{\rm{f}}}}}},{{{{{\rm{c}}}}}}}$ values determined by classical analytical fit (orange dots) and global ${E}_{a}$ fit (black dots) from curves exemplarily shown in **A**. The corresponding linear regressions are represented by the dashed lines. **C** ${E}_{a}$ values of AQP1 (dots) determined from the slopes shown in the middle graph with standard deviation (filled area) and mean values (dashed lines) for classical analytical fit (orange) and global ${E}_{a}$ fit (black). Standard error of the mean and the confidence interval in dependence of the number of measurements is depicted in Fig. S6.

As outlined above, accurate *E*_*a*_ estimation relies on a large number of experiments performed for a wide temperature range with samples exhibiting a high protein density in the vesicle preparation when using a traditional data evaluation approach. These prerequisites were hardly met in the past. Here, we show that utilizing a revised data evaluation procedure based on a global analytical ${E}_{a}$ fit to stopped-flow data changes the situation. It enables us to get accurate ${E}_{a}$ values with a reasonable number of experimental repetitions and realistic reconstitution efficiencies. The insights obtained from our analysis were used for accurate ${E}_{a}$ estimation of AQP1. To this end, we overexpressed, purified and reconstituted AQP1 into large unilamellar vesicles. Subsequently, these vesicles were subjected to a hyperosmotic solution at different temperatures in a stopped-flow apparatus (SFM-300 and µ-SFM, Bio-Logic, Claix, France). Exemplary scattering data are shown for empty bare lipid vesicles and PLs in Fig. 4A. We fitted these datasets with our global ${E}_{a}$ fit shown as dashed black lines in Fig. 4A. For comparison, we analyzed these data separately with a traditional analytical fit. In both cases the ${P}_{f,m}$ was kept shared during data fitting. Figure 4B summarizes the results of 3 independent purifications and 14 independent reconstitutions. Orange dots depict ${P}_{f,c}$ values from the classical analytical fits to each sample measured at corresponding temperatures. Each reconstitution series was fit to the line equation to calculate the corresponding ${E}_{a}$ values summarized in Fig. 4C. As can be seen the variation of the ${E}_{a}$ values was large (±13 kcal/mol). In contrast, the black dots in Fig. 4B show a perfect linear dependence due to the global ${E}_{a}$ fitted to the data. The ${E}_{a}$ values extracted from the fits vary between 2.9 and 4.5 kcal/mol (Fig. 4C) with an average of 3.75 ± 0.16 kcal/mol (Supplementary Fig. 6). This is well within the scatter of reported ${E}_{a}$ values for AQP1 (Supplementary Figure 1) and less than the ${E}_{a}$ for self-diffusion of water molecules in the bulk (~4.6 kcal/mol)^17^.

### Entropic barrier of water permeation through AQP1

**Fig. 5:**
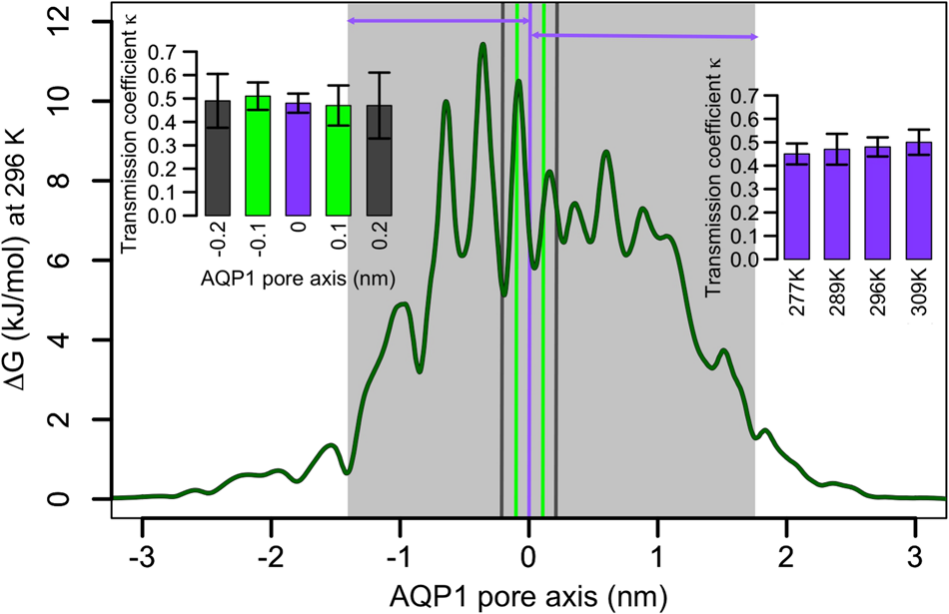
Determination of the transmission coefficient *κ* from MD simulations of AQP1. The free energy profile of water molecules passing the pore of AQP1 at 296 K (shown as dark green line in the main plot) was used to determine the borders of the pore to be at -1.4 and 1.7 nm (shaded area). The different positions of the dividing surface ${{{{{{\rm{z}}}}}}}_{{{{{{\rm{div}}}}}}}$ are visualized by vertical bars (purple at 0 nm, green at -0.1 and at 0.1 nm, and dark gray at -0.2 and 0.2 nm). The transmission coefficients ${{{{{\rm{\kappa }}}}}}$ determined for this variation of ${{{{{{\rm{z}}}}}}}_{{{{{{\rm{div}}}}}}}$ at 296 K are shown in left inset. The inset on the right side shows the differences of $\kappa$ for dividing surface ${z}_{{div}}$ = 0 nm for simulations performed at different temperatures. The error bars denote the standard deviations over the four individual pores and over the two halves of the channel.

According to Eqs. (1)–(3) the entropy of activation is accessible via a combination of accurate ${E}_{a}$ values as presented herein and accurate ${p}_{f}$ values which we published in 2015^5^, or in other words, via temperature dependent rates of water permeation through AQP1. Combining Eqs. (2) and (3), we obtain

6 $${p}_{f}={\nu }_{0}{v}_{w}{e}^{1+\triangle {S}^{{{\ddagger}} }/R}{e}^{-{E}_{a}/{RT}}=A{e}^{-{E}_{a}/{RT}}$$

where ${v}_{w}=\frac{{V}_{W}}{{N}_{A}}$ is the molecular volume of water and $A={\nu }_{0}{v}_{w}{e}^{1+\triangle {S}^{{{\ddagger}} }/R}$ the pre-exponential factor that we retrieved by the fitting routine presented above. *A* can be interpreted as the maximal rate of water flux in the absence of any activation barrier of water permeation, but an entropic difference of the transition state compared to the water in bulk only. Equation (6) is derived from transition-state theory (TST), in which a transition between two states occurs every $1/{\nu }_{0}$ in the absence of a barrier. However, *ν*_0_ represents the upper limit for a true transition rate, which in practice is reduced as not every barrier crossing results in a transition event. To account for these so-called re-crossing events, and in accordance with modifications to the classical TST^34,35^, we introduced the transmission coefficient *κ*, such that ${A}^{{\prime} }=\kappa {\nu }_{0}{v}_{w}{e}^{1+\triangle {S}^{{{\ddagger}} }/R}=\kappa A$ ^36^ and determined its value using molecular dynamics simulations to be ${\kappa }_{{AQP}1}$ = 0.48 ± 0.04 for AQP1. The value can be assumed to be constant within the experimental temperature range (Fig. 5) and does only effect $\triangle {S}^{{{\ddagger}} }$ but not $\triangle {H}^{{{\ddagger}} }$.

**Fig. 6:**
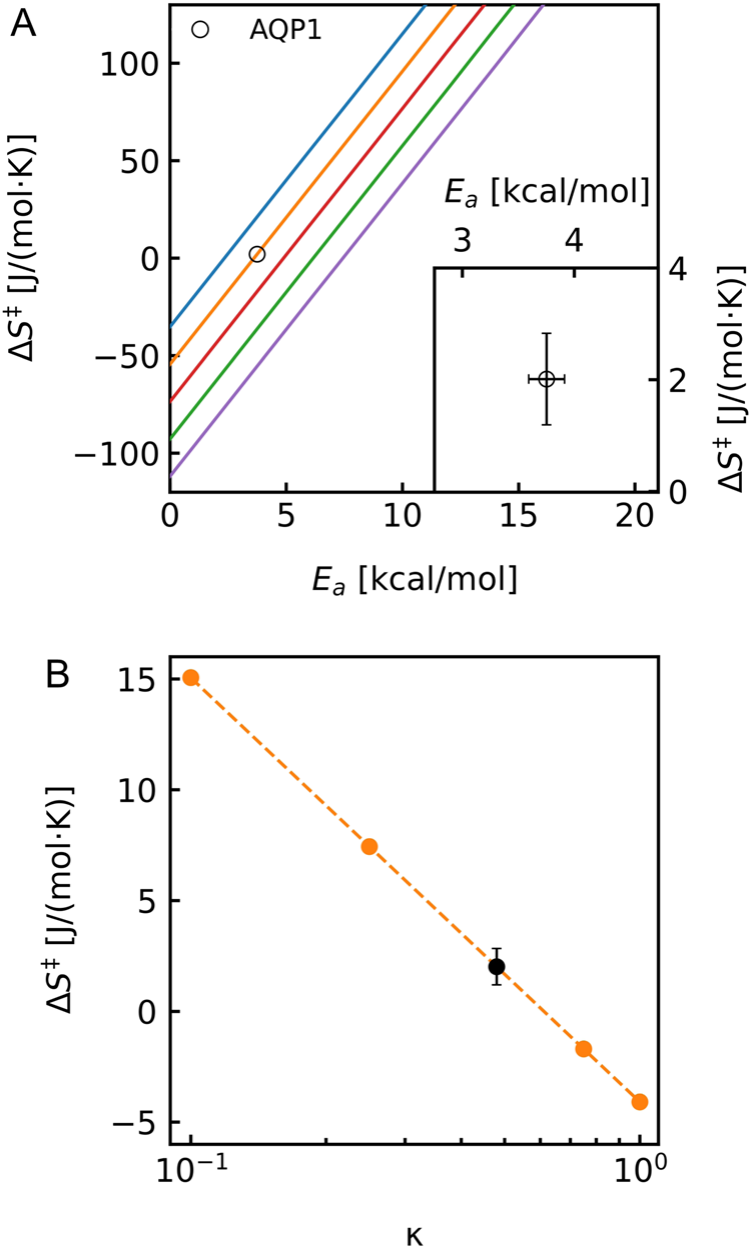
Entropy of activation over activation energy. **A** $\triangle {S}^{{{\ddagger}} }$ plotted over ${E}_{a}$ with a ${p}_{f}$ of $3.25\cdot {10}^{-12}$ (blue line), $3.25\cdot {10}^{-13}$ (orange line), $3.25\cdot {10}^{-14}$ (red line), $3.25\cdot {10}^{-15}$ (green line) and $3.25\cdot {10}^{-16}$ ${{cm}}^{3}/s$ (purple line). Black ring represents the here determined value of 2.01 ± 0.82 J/(mol·K) for AQP1 with error bars shown zoomed-in as an inset. **B** $\triangle {S}^{{{\ddagger}} }$ plotted for different *κ*. Orange dots illustrate theoretical values of *κ* ranging from 0.1 to 1 ($\triangle {S}^{{{\ddagger}} }$ = 15.05 J/(mol·K), 7.44 J/(mol·K), −1.7 J/(mol·K), −4.09 J/(mol·K) for *κ* = 0.1, 0.25, 0.75, 1, respectively). The black dot shows $\triangle {S}^{{{\ddagger}} }$ = 2.01 ± 0.82 J/(mol·K) for $\kappa =0.48$ of AQP1 determined here by MD simulations.

Using ${A}^{{\prime} }$ enables us to calculate the first experimentally determined entropic barrier for water permeation through AQP1, as a representative of narrow biological single-file channels. ${\triangle {S}^{{{\ddagger}} }}_{{AQP}1}$ amounts to 2.01 ± 0.82 J/(mol·K), which contributes 0.14 ± 0.05 kcal/mol to the free energy at 298 K. This value is 2.3 times larger and opposite in sign to the only available estimate of $\triangle {S}^{{{\ddagger}} }$ in the literature (i.e., **–**0.87 J/(mol·K)). The latter was derived from a semilogarithmic plot of the effective water permeability ${P}_{f}$ of various types of lipid bilayers and single-file channels as a function of *E*_*a*_^37^. Yet, the validity of this approach is questionable as it lumps together water permeation through vastly different types of channels and lipid bilayers. If $\triangle {{{{{{\rm{S}}}}}}}^{{{\ddagger}} }$ can be assumed to be similar for such disparate diffusion processes requires verification in the future. Regardless, Fig. 6A illustrates that to allow mathematical compatibility of high ${p}_{f}$ and high ${E}_{a}$ values (as reported in the experimental literature for artificial water channels^10–14^), $\triangle {S}^{{{\ddagger}} }$ would have to adopt values larger than 50 J/(mol·K). Such values seem unrealistic for AQP1 even if our estimation of *κ* would be significantly off (Fig. 6B).

## Discussion

We obtained precise estimates for ${E}_{a}$ values of water permeation through water channels with the stopped-flow methodology by capitalizing on high reconstitution efficiencies, several rounds of independent measurements and our global ${E}_{a}$ fitting routine. The latter increases the attainable precision by exploiting the fact that (i) the fraction of protein-free vesicles and (ii) ${E}_{a}$ are equal for the same vesicle population. For this reason, both, the empty vesicle fraction and ${E}_{a}$ can be treated as global fit parameters. The global fits yield a low permeability ${P}_{f,m}$ for the background permeability of the lipid bilayer (i.e., slow component) and a high permeability ${P}_{f,c}$ for the permeability of the reconstituted water channels (i.e., fast component). However, an accurate *E*_*a*_ estimation relies on the ability to separate the two permeabilities. Separation is difficult when the membrane permeability is high. Specifically, if the channel has an inherently low permeability or if the channel density is low. Hence, if for technical reasons, measurements in highly permeable membranes cannot be avoided, the reconstitution efficiency needs to be improved. The reduction of the background permeability is an alternative approach to increase the resolution. Possible strategies to reduce the background permeability include elevated concentrations of sterols, e.g. cholesterol, or a decreased membrane fluidity^19^. Yet, as explicated below an altered lipid composition can affect channel function. Depending on the impact of the lipid bilayer composition on protein function similar considerations as above apply (i.e., a decrease in channel permeability reduces separability. For an increase the opposite is the case.).

The lipid composition can affect the water permeability in our system in multiple ways. It can influence the (i) background permeability through the lipid bilayer, (ii) the structure/function relation of the reconstituted membrane protein, and (iii) the reconstitution efficiency into the PLs. Thereby, the hydrophobic mismatch between lipids and proteins impinges on protein orientation, aggregation, and functionality^38–40^. Moreover, interfacial lipids stabilize membrane protein oligomers^41^ and they can change protein structure and function^42,43^. The effects lipids exert on proteins can result either from specific binding^44–46^ or from changes in membrane properties such as fluidity, hydrophobic thickness, surface charge, curvature, and surface tension^47,48^. Also, the function of aquaporines was shown to depend on the lipid composition: For example, it was demonstrated that the water permeability of AQP4 depends on membrane compressibility and thickness^47^. Similarly, the ribitol transport capability of GlpF, an aquaglyceroporin of E. coli, was shown to be modulated by negatively charged lipids^49^. In the case of AQPZ, cardiolipin was found to bind preferentially to the contact site of monomers in the tetrameric structure^50^ and to increase its activity^42^. We previously showed that cardiolipin also binds into the groove between adjacent AQP1 monomers^18^. Furthermore, we identified several negatively charged phosphatidyl glycerol lipids, which interact with positively charged amino acids at the interface between the lipid and AQP1. Hence, it is conceivable that the function of AQP1 is also regulated by specific protein-lipid interactions. If true, measurements at various lipid compositions might give different ${p}_{f}$ and ${E}_{a}$ values. Depending on the accompanying structural changes this may or may not affect the entropic barrier of water permeation through AQP1.

The appropriate temperature range must also be carefully chosen. It ought to be as large as possible but at the same time allow to distinguish the kinetics of water flow through PLs from that through protein-free vesicles. Because the ${E}_{a}$ value of water permeation through the lipid bilayer is larger than that of facilitated transport through channels, the difference in the rate of water flow vanishes at higher temperatures. However, narrowing the experimental temperature range, e.g. to the low temperature regime, reduces the accuracy of the ${E}_{a}$ estimation as shown in Fig. 2. For AQP1 reconstituted into PLE liposomes, we chose a range between 277 K and 301 K with at least 4 equidistant temperatures. We determined ${E}_{a}$ of water permeation through AQP1 to be 3.75 ± 0.16 kcal/mol from five sets of stopped-flow measurements of independent purifications and subsequent reconstitutions with 14 PL samples. We obtained an estimate for the entropy of activation of AQP1 of 2.01 ± 0.82 J/(mol·K) by relying (i) on our measurements of ${P}_{f,c}$ at different temperatures (ii) on our recently published single channel permeability *p*_*f*_ of 3.20 10^-13^ cm^3^/s at 278 K^5^, and (iii) on our efforts to determine the transmission coefficient (*κ*) by utilizing MD simulations. (i.e., *κ* = 0.48 ± 0.04*)*. The entropic component contributes 0.14 ± 0.06 kcal/mol to the free energy $\triangle {G}^{{{\ddagger}} }$ at 298 K. In contrast, $\triangle {H}^{{{\ddagger}} }$ contributes 3.16 ± 0.16 kcal/mol to $\triangle {G}^{{{\ddagger}} }$ of 3.02 ± 0.22 kcal/mol at this temperature. We note that $\triangle {S}^{{{\ddagger}} }$ and $\triangle {H}^{{{\ddagger}} }$ can also be obtained from the linearized form of the Eyring equation (Supplementary Note 1), where the two parameters can be directly extracted from the slope and the y-intercept of the linear fit.

Comparison with other channels is difficult due to the low number of published quantitative *p*_*f*_ and *E*_*a*_ values^16^ and the undetermined values of *κ*. However, our ${\kappa }_{{AQP}1}$ of 0.48 ± 0.04 at 296 K is in line with literature values of other processes in the same temperature regime. Literature *κ* values are between to 0.3-0.9^51,52^ for reactions such as the 1,4-hydrogen shift isomerization of the 2-cyclohexylethyl radical in the gas phase, 0.5 – 1 for enzymatic reactions^34^, 0.1 for DNA intercalation^53^ and protein folding^54^. In general, however, values for $\kappa$ are thought to be close to 1^55^. With ${\kappa }_{{AQPZ}}$ = 0.48 ± 0.05 at 296 K and ${\kappa }_{{CNT}}$ = 0.51 ± 0.02 at 299 K we find similar values for AQPZ and nCNTPs (Supplementary Figure 7). This suggests minor variations between AQPs in particular and single-file channels in general. AQPs exhibit a conserved fold of the single-file region^56^. H-bond forming residues (i.e., in total 13 potential H-bonding sites^5,56^ are lined up on one side of the channel, except at the selectivity filter region. For this reason, water molecules are rotationally^57^ and translationally^5^ restricted. The structural similarity of AQPs suggest a similar energetic contribution for all AQPs. However, we recently showed, that the dehydration penalties for water molecules entering the single-file region of AQP1, AQPZ, and AQP4 differ^58^. This might affect the enthalpic component of the barrier and thus lead to a slightly altered activation energy of AQPZ and AQP4 as compared to that of AQP1. The single-file pore architecture of other channels can be quite different^6^. For example, the selectivity filter of the bacterial potassium channel KcsA is constituted by 20 symmetrically arranged carbonyl groups^5^. This distribution is expected to allow for larger rotational freedom of water molecules in the pore of KcsA compared to AQPs. In narrow nCNTPs the water molecules are not rotationally restricted. For this reason, they exhibit higher rotational dynamics, especially at low temperatures^59^. The enhanced rotational freedom and the corresponding increase in entropy can explain the lower Gibb’s free energy barrier for water permeation through nCNTPs as compared to the one in AQPs. Yet, this awaits experimental verification in the future.

## Conclusion

Our analysis provides a starting point to understand (i) the discrepancy between the reported values for $\triangle {S}^{{{\ddagger}} }$ for biological and artificial channels, (ii) the effect of the pore geometry, and (iii) of channel lining residues on $\triangle {S}^{{{\ddagger}} }$. In addition, the numeric values of $\triangle {S}^{{{\ddagger}} }$ and $\triangle {H}^{{{\ddagger}} }$ can provide insights into the nature of the investigated process. This information may open new avenues for the design of entropically vs enthalpically driven systems/devices.

## Methods

### Protein expression, purification, and reconstitution

AQP1 was expressed, purified, and reconstituted as previously described^5^. *S. cerevisiae* strain InvSc1 (Invitrogen) transfected with pYES2 His-YFP-hAQP1 was grown in DOB-Ura and transferred to YPG for induction of gene expression at an OD600 of 2.0. Cells were harvested after 16 h at 303 K by centrifugation. Cell pellets were resuspended in lysis buffer (100 mM KPh Buffer, pH8; cOmplete protease inhibitor cocktail, EDTA-free, Roche) and disrupted using 3 rounds of 20000 psi by EmulsiFlex (Avestin). The lysate was cleared for 10 min at 6,000 × g and 277 K, followed by an ultracentrifugation for 2 h at 120,000 × g and 277 K. Next, membrane pellets were homogenized in solubilization buffer (100 mM KPh pH 8, 200 mM NaCl, 10% glycerol, 5 mM β-mercaptoethanol, 20 mM imidazole) in the presence of 3% OG for 1 h at 277 K. After ultracentrifugation for 1 h at 120,000 × *g* and 277 K supernatants were incubated with equilibrated Ni-NTA resin overnight at 277 K. Ni-NTA resin was washed with 100 CV using wash buffer (100 mM KPh pH 7.5, 200 mM NaCl, 10% glycerol, 5 mM β-mercaptoethanol, 100 mM imidazole) in the presence of 2% OG. Elution of His-YFP-AQP1 was performed using elution buffer (100 mM KPh pH 7, 200 mM NaCl, 10% glycerol, 5 mM β-mercaptoethanol, 1 M imidazole).

For protein reconstitution, *E.coli* polar lipids (PLE, Avanti Polar Lipids, Alabaster, AL, USA) were dissolved in chloroform and doped with Atto633PPE. Subsequently, the suspension was dried on a rotary evaporator for ~ 1 h. The dry lipid film was rehydrated with reconstitution buffer (100 mM NaCl, 10 mM MOPS, 1.33 % OG, pH 7.4) to a final lipid concentration of 20 mg/ml and bath sonicated for 10 – 15 min. Afterward, the vesicle suspension was mixed with the purified protein and incubated for 1 h at room temperature. In 3 steps with increasing amount of Bio-Beads SM-2 resin (Bio-Rad Laboratories, Hercules, CA, USA) the detergent was removed at 277 K within 48 h. Proteoliposomes (PL) were pelleted at 100,000 × *g* for 100 min at 277 K and resuspended in reconstitution buffer without OG. Finally, the sample was extruded through two polycarbonate filters with 100 nm pore sizes and assayed without delay.

### *E*_*a*_ estimation using LUVs and stopped-flow data

Stopped-flow experiments have been performed as described previously^22^. In brief, LUV’s were subjected to hyperosmotic buffer (100 mM NaCl, 10 mM MOPS, 150 mM sucrose) in a stopped-flow apparatus (SFM-300 or µ-SFM, Bio-Logic, Claix, France). Scattered light intensity of 546 nm wavelength was detected at an angle of 90°. The resulting intensity traces were normalized by ${I}_{{norm}}(t)=(I\left(t\right)-{I}_{\min })/({I}_{\max }-{I}_{\min })$, where $I(t)$ is the measured intensity at time *t* and ${I}_{\max }$ and ${I}_{\min }$ are the averaged maximum and minimum intensities. $I(t)$ is related to the vesicle volume $V(t)$ with coefficients *a* and *b* by $I\left(t\right)=a-b\cdot V\left(t\right)$ and correspondingly

7 $${I}_{{norm}}\left(t\right)=1-b\cdot V\left(t\right)$$

The vesicle shrinkage is given by

8 $$\frac{{dV}(t)}{{dt}}=S{P}_{f}{V}_{w}\left({c}_{{in}}(t)-{c}_{{out}}\right)$$

9 $${c}_{{in}}\left(t\right)=\frac{{V}_{0}}{V(t)}{c}_{{in},0}$$

where *S*, *P*_*f*_, *V*_*w*_, ${c}_{{in},0}$ and ${c}_{{out}}$ are the surface of the vesicle, the water permeability of the membrane, the molar volume of water, the initial osmotic concentration inside the vesicle, and the osmotic concentration of the external solution.

For the classic analytical fit to extract *P*_*f*_ from $V\left(t\right)$ we use our recently published analytical solution to Eq. 8(*5*)

10 $$V\left(t\right)={V}_{0}\frac{{c}_{{in},0}}{{c}_{{out}}}\left\{1+L\left(\frac{{c}_{\varDelta }}{{c}_{{in},0}}\exp \left(\frac{{c}_{\triangle }}{{c}_{{in},0}}-\frac{S{P}_{f}{V}_{w}{{c}_{{out}}}^{2}}{{V}_{0}{c}_{{in},0}}t\right)\right)\right\}$$

where *L* is the Lambert function, defined by $L\left(x\right){e}^{L\left(x\right)}=x$ and ${c}_{\triangle }={c}_{{out}}-{c}_{{in},0}$. To take into account the fraction *X*, which is the fraction of protein-free vesicles, the total volume is introduced

11 $${V}_{{tot}}\left(t\right)={X\cdot V}_{{lip}}\left(t\right)+(1-X){\cdot V}_{{prot}}\left(t\right)$$

where ${V}_{{empty}}$ and ${V}_{{prot}}$ are the volume of the empty vesicles and the volume of the PLs. Another approach to determine ${P}_{f}$ is an exponential fit, which uses the following relation between ${P}_{f}$ and the time constant *τ*^21^

12 $${P}_{f}=\frac{{r}_{0}}{3{V}_{w}\tau }\frac{{c}_{{in},{0}}+{c}_{{out}}}{2{c}_{{out}}^{2}}$$

where *τ* can be extracted by fitting the scattered intensity with an exponential function of the form

13 $$I\left(t\right)={I}_{0}-a \cdot {{\exp }}\left(-\frac{t}{{\tau }_{{empty}}}\right)-b\cdot {{\exp }}\left(-\frac{t}{{\tau }_{{prot}}}\right)$$

where ${\tau }_{{empty}}$ and ${\tau }_{{PL}}$ are the time constants for the protein-free vesicles and the proteoliposomes.

For the evaluation of the activation energy ${E}_{a}$ either an Arrhenius plot, where the natural logarithm of ${P}_{f,c}$, determined with a classic analytical fit by using Eqs. (7), (11), and (10) or an exponential fit (Eq. 13), is plotted over $1/T$ (in $[{K}^{-1}]$), or a global ${E}_{a}$ fit has been performed. Regardless of which fitting routine is applied, ${P}_{f,m}$ is fixed to the protein-free control sample in order to track the empty fraction. For the global ${E}_{a}$ fit, the ensemble of curves of a sample at different temperatures is fitted globally using the analytical solution (Eq. 10). Instead of evaluating ${P}_{f}$‘s for the different temperatures individually and independently, the permeability values were substituted by the following expression exploiting Eqs. (4) and (5):

14 $${P}_{f}(T)=A{e}^{-\frac{{E}_{a}}{{RT}}}+{P}_{f,m}(T)$$

All osmotic water flux measurements of a sample recorded at different temperatures were simultaneously fitted using Eqs. (7), (10), (11), and (14). Here, the only free fit parameter, which was evaluated for the different temperatures independently is *b* from Equation 7, whereas the global variables ${E}_{a}$, $A$ and $X$ are equal for all datasets of an ensemble.

### GUV formation

GUVs were formed as described previously^33^ using the electro-formation method. Briefly, 5 µl of reconstituted AQP1 PL solution were placed as small droplets onto two parallel platinum wires (PT005157 Platinum Wire, Goodfellow Cambridge Ltd. Huntington, England), with a length of 40 mm, a diameter of 1 mm and an inner edge distance of 3 mm. After applying a gentle stream of argon for 15–30 min the partially dehydrated lipid film was rehydrated in GUV formation buffer (5 mM KCl, 1 mM HEPES, pH 7.3 and sucrose to a final osmotic concentration of 450 mOsm). Subsequently, a 10 Hz sine wave with 64 equal steps raising the peak-to-peak voltage from 50 to 704 mV every 28 s followed by additional 2 h at 704 mV was applied using a function generator (HP Agilent 33120 A function generator, Agilent Technologies Inc., Santa Clara, CA, USA; set to 50 Ω termination).

### Micropipette aspiration methodology

Microaspiration pipettes filamentless borosilicate glass capillaries (GB150-10; Science Products GmbH, Hofheim, Germany) were pulled, bent to an angle which allows parallel alignment of the pipette tip to the focal plane of the microscope and broken to an inner tip diameter of 5 to 8 µm. The pipettes were filled with GUV bath solution (5 mM KCl, 5 mM HEPES, pH 7.2, and glucose to 450 mOsm) with additionally 1 mg/ml Bovine serum A (BSA) and attached to the micromanipulator (Scientifica PatchStar; Scientifica Ltd, East Sussex, United Kingdom). With a second micromanipulator (Eppendorf Patchman 2), with an additional step motor (SF-77B; Warner Instruments, Hamden, CT, USA) which allowed rapid switching between the barrels, a multibarrel square glass capillary (SG-800/5 Warner instruments, Hamden, CT, USA) was placed in close vicinity to the aspirated GUV and perfusion was applied. Equatorial plane LSM images (LSM 510 META; Carl Zeiss, Jena, Germany) monitored the volume change of the GUVs upon perfusion with hyperosmotic buffer (5 mM KCl, 5 mM HEPES, pH 7.2, glucose to 450 mOsm and additionally 80 mM sucrose). GUV deflation was analysed as previously described^33^. Representative fits to the raw data are shown in Supplementary Figure 8. To control the temperature, the perfusion pipette, the GUV bath solution chamber and the objective of the microscope were water cooled. After every measured GUV, the temperature used for the Arrhenius plot was taken close to the aspiration pipette’s position. Confocal volume estimation and pinhole calibration were done with Rhodamin 6 G (Invitrogen, San Diego, CA).

### Protein counting with fluorescence correlation spectroscopy (FCS)

FCS was used to determine the channel density within the GUV membrane as previously described in Ref. ^33^. In brief, the number of YFP-labeled AQP1 was derived from the autocorrelation function ($G(\tau )$) of the temporal fluorescence intensity signal, detected with a commercial laser scanning microscope (LSM 510 META ConfoCor 3 with a 40x-UOPLAN water immersion objective; Carl Zeiss, Jena, Germany) using avalanche photodiodes.

### *E*_*a*_ estimation using GUVs and the micropipette aspiration methodology

Image analysis (Fiji distribution, ImageJ)^60^ tracked changes in the vesicle contour and therefore in the GUV’s volume. This volume change was analyzed by fitting with the analytical solution, described in detail elsewhere^33^.

### *E*_*a*_ modeling

For simulating the dependency of the error in *E*_*a*_ on the error in ${P}_{f}$ we simulated Gauss-distributed ${P}_{f}$ values for each of 5 temperatures specified in the corresponding plots with a standard deviation *σ* of 5%, 10%, 20%, and 30%, respectively. Subsequently, an Arrhenius plot has been performed and the activation energy has been determined from the slope. Overall, 10,000 *E*_*a*_ values have been simulated and the average of n determined ${E}_{a}$’*s* has been plotted as shown for instance as dots in Figs. 1 and  2.

### Transmission coefficient *κ* determination

To extract $$\triangle {S}^{{{\ddagger}} }$$ from the pre-exponential factor of the $${{{{{\rm{ln}}}}}}({p}_{f}\left(T\right))$$ fits, the transmission coefficient *κ* was introduced. The value for *κ* was determined using all-atom molecular dynamics (MD) simulations in which the total amount of water molecules passing the pore (the pore exits/entries determined from the free energy profile of water molecules in the pore are located at −1.4 nm and 1.7 nm) from both sides $${N}_{{{{{{\rm{through}}}}}}}$$ was set into relation with water molecules making it half way through the pore $${N}_{{{{{{\rm{half}}}}}}-{{{{{\rm{way}}}}}}}$$, $$\kappa ={N}_{{{{{{\rm{half}}}}}}-{{{{{\rm{way}}}}}}}/$$$${N}_{{{{{{\rm{through}}}}}}}$$. In particular, $${N}_{{{{{{\rm{half}}}}}}-{{{{{\rm{way}}}}}}}$$ was defined by crossing the position $${z}_{{{{{{\rm{div}}}}}}}$$. Varying $${z}_{{{{{{\rm{div}}}}}}}$$ in the range from −0.2 till 0.2 nm similar *κ* values were estimated, yet with different standard deviations over the halves of the pores and individual chains. In detail, *κ* = 0.49 ± 0.12 at $${z}_{{{{{{\rm{div}}}}}}}$$ = −0.2 nm, 0.51 ± 0.06 at $${z}_{{{{{{\rm{div}}}}}}}$$ = −0.1 nm, 0.48 ± 0.04 at $${z}_{{{{{{\rm{div}}}}}}}$$ = 0 nm, 0.47 ± 0.09 at $${z}_{{{{{{\rm{div}}}}}}}$$ = 0.1 nm and 0.47 ± 0.14 at $${z}_{{{{{{\rm{div}}}}}}}$$ = 0.2 nm. The smallest deviations appeared to be at $${z}_{{{{{{\rm{div}}}}}}}=0$$ nm, thus the temperature dependence of *κ* was determined at $${z}_{{{{{{\rm{div}}}}}}}=0$$ nm. The resulting $$\kappa$$ values amount to 0.45 ± 0.05 at 277 K, 0.47 ± 0.07 at 289 K, 0.48 ± 0.04 at 296 K and 0.50 ± 0.05 at 309 K. Thus, for AQP1 the transmission coefficient is largely independent of the temperature and of the position of the dividing surface and the average value $$\kappa =0.48$$ lies within the expected range found in literature^34,51,52^.

### MD simulations

All MD simulations were performed using the CHARMM36(m) force field^61–63^ and TIP4p water model^64^ in GROMACS 2020^65^. The AQP1 and AQPZ systems containing tetrameric AQPs embedded in an *E. coli* polar lipid extract membrane mimic^18^ were taken from our previous works^56^ and were simulated at 277 K, 289 K, 296 K and 309 K. The structure and the simulation parameters for the nCNTP were generated by the nanomaterial builder^66^ of CHARMM-GUI^67^ and converted to GROMACS by psf2itp.py. Thereby, the diameter and the symmetry^6^ of the single-file nCNTPs were based on previous work^68^, but the nanotube consisted of 16 repetitions along its pore axis, to reach a membrane-spanning length of 4 nm. The nCNTP was then embedded into a small patch (86 lipids per leaflet) of 1,2-dilauroyl-sn-glycero-3-phosphocholine (DLPC) bilayer, due to its small thickness and low phase transition temperature of 271 K. The DLPC membrane patch comprising 90 lipids in each leaflet was prepared using our multiscaling methodology^69^. In short, the bilayer was generated by insane^70^, equilibrated at coarse-grained resolution using the Martini2 force field^71^ and non-polarizable water, and converted back to all-atom resolution of CHARMM36^62^ by backward^72^. For nCNTP insertion a hole in the membrane was prepared by deleting 4 DLPC in each leaflet. After multiplication in the membrane plane, the DLPC membrane containing 4 individual nCNTPs was solvated by about 31 000 waters, energy minimized by 1000 steps of steepest descent and equilibrated at 309 K, while keeping the x and y positions of the nCNTPs position restrained. This allowed for movement along the membrane normal, together with the membrane, but to avoid tilting of the nCNTPs and eventual closure of the pore by full insertion into the lipid bilayer. nCNTPs were simulated at 289 K, 299 K, 309 K, and 319 K.

All production run MD simulations were 500 ns long, using the time step of 2 fs and saving the trajectory files every 10 ps. The water flux was monitored by g_flux^73^ individually for each chain. Afterward, the wrongly identified water transition events were sorted out by a home-written script. The temperature, T, specific free energy of water permeation, $\triangle G\left(z\right)$, was calculated using

15 $$\triangle G\left(z\right)=-{RT}\cdot {{{{\mathrm{ln}}}}}(d)$$

based on the normalized distribution, *d*, of water molecules along the pore axis, *z*. The distribution was estimated by counting the number of water oxygens in 0.05 nm-sized bins along the z axis in a 1.5 × 1.5 × 6 nm cuboid centered at the center of mass of the pore.

### Error propagation of $\triangle {S}^{{{\ddagger}} }$

To estimate the error $\delta \left(\triangle {S}^{{{\ddagger}} }\right)$ on the value of the entropic barrier $\triangle {S}^{{{\ddagger}} }$ the errors $\delta \left({p}_{f}\right)$, $\delta \left(-{E}_{a}\right),\delta \left(\kappa \right)$ are propagated. One can write

16 $${e}^{1+\triangle {S}^{{{\ddagger}} }/R}={p}_{f}{e}^{-{E}_{a}/{RT}}/({\nu }_{0}{v}_{w}\kappa )$$

which yields

17 $${{{{{\rm{\delta }}}}}}\left({e}^{1+\triangle {S}^{{{\ddagger}} }/R}\right)={e}^{1+\triangle {S}^{{{\ddagger}} }/R}\sqrt{{{{{{{\rm{\delta }}}}}}\left({p}_{f}\right)/{p}_{f}}^{2}+{({{{{{\rm{\delta }}}}}}\left(-{E}_{a}\right)/{RT})}^{2}+{(-{{{{{\rm{\delta }}}}}}\left(\kappa \right)/(\kappa ))}^{2}}$$

The relation ${{{{{\rm{ln}}}}}}\left({e}^{1+\triangle {S}^{{{\ddagger}} }/R}\right)-1=\triangle {{{{{{\rm{S}}}}}}}^{{{\ddagger}} }/R$ leads to

18 $${{{{{\rm{\delta }}}}}}\left(\triangle {{{{{{\rm{S}}}}}}}^{{{\ddagger}} }\right)=R\frac{{{{{{\rm{\delta }}}}}}\left({e}^{1+\triangle {S}^{{{\ddagger}} }/R}\right)}{{e}^{1+\triangle {S}^{{{\ddagger}} }/R}}=R\sqrt{{{{{{{\rm{\delta }}}}}}\left({p}_{f}\right)/{p}_{f}}^{2}+{({{{{{\rm{\delta }}}}}}\left(-{E}_{a}\right)/{RT})}^{2}+{(-{{{{{\rm{\delta }}}}}}\left(\kappa \right)/(\kappa ))}^{2}}$$

For AQP1, this amounts to 0.82 J/(mol·K), where the biggest contribution to this uncertainty lies within the varying ${p}_{f}$ followed by the uncertainty for $\kappa$. In comparison, the error in ${E}_{a}$ is negligible.

## Supplementary information

Supplementary Information

## Data Availability

The datasets generated during and/or analyzed during the current study are available from the corresponding author on reasonable request.

## Code availability

Data used to perform MD simulations have been deposited in the DaRus repository under the link 10.18419/darus-3390. The Python code used for global activation energy fits is deposited in the Zenodo repository under the DOI 10.5281/zenodo.7848302.
