# Supplementary material for: Entropic barrier of water permeation through single-file channels
Source: Commun Chem. 2023 Jun 29;6:135. doi: 10.1038/s42004-023-00919-0 (PMC10310842; doi:10.1038/s42004-023-00919-0)
Supplement: Supplementary file 1 — Supplementary Information [file 42004_2023_919_MOESM1_ESM.pdf]

Supplementary Information

Entropic barrier of water permeation through single-file channels

Johann Wachlmayr[1], Gotthold Flaeschner[2], Kristyna Pluhackova[3], Walter Sandtner[4], Christine Siligan[1] and Andreas Horner[1,#]

[1]Institute of Biophysics, Johannes Kepler University Linz, Gruberstr. 40, 4020 Linz, Austria; [2]Department of Biosystems Science and Engineering, Eidgenössiche Technische Hochschule (ETH) Zürich, Mattenstr. 26, 4058 Basel, Switzerland; [3]Stuttgart Center for Simulation Science, Cluster of Excellence EXC 2075, University of Stuttgart, Stuttgart, Germany; [4]Center of Physiology and Pharmacology, Institute of Pharmacology, Medical University of Vienna, Vienna, Austria;

[#]Correspondence should be sent to andreas.horner@jku.at

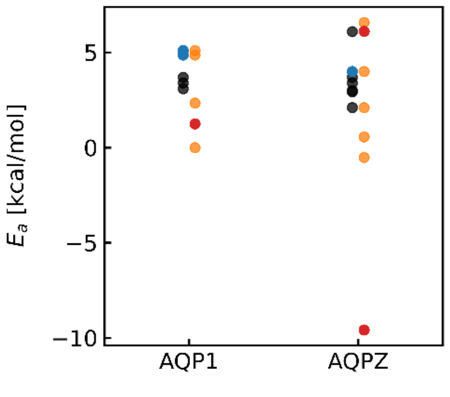

Supplementary Figure 1. Scatter of published literature values. *Literature values of E_a for AQP1 (1-5) and AQPZ (6-11) (black and blue dots). Blue dots (2, 3, 10) indicate values which were calculated using background correction. To emphasize the effect of background correction we also corrected the values visualized as black dots. The respective values after background correction are shown in orange and red color. While red dots indicate literature values which have not been background corrected, for the other black labeled values there are no indications in the respective publication (neither background correction is mentioned nor evident from the published data) in favor of a background correction.*

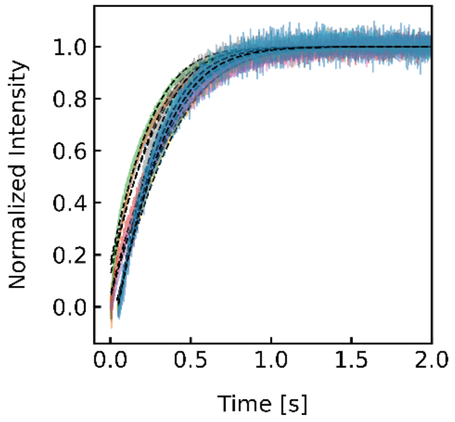

P_f (µm/s)	d (nm)
6.17114	80.2
6.890632	83.2
6.805071	76.4
5.987022	89.7
6.361547	105.0
7.111855	114.7
6.795241	99.6
8.794411	100.8
8.40217	106.8
7.723728	105.7

Supplementary Figure 2. Standard deviation of stopped-flow measurements. *Water flux measurements of PLE liposomes (100 mM NaCl, 10 mM MOPS, pH 7.4) exposed to hyperosmotic gradient (150 mM sucrose, 100 mM NaCl, 10 mM MOPS, pH 7.4). 10 curves, where each represents the average of 7 to 9 stopped-flow curves, are plotted and fitted with the analytical solution, subsequently. The results are shown in the table with an average of 7.1 ± 0.93 µm/s which corresponds to a standard deviation of 13%. The diameter d of the vesicle population was estimated via dynamic light scattering as previously described (12).*

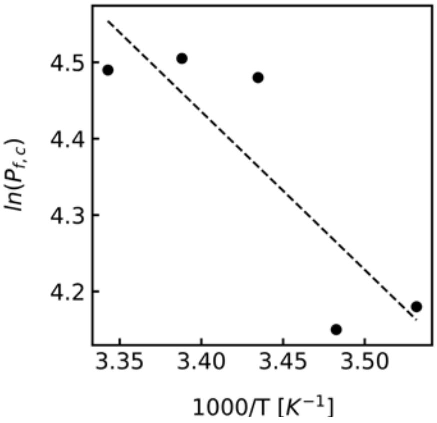

Supplementary Figure 3. Arrhenius plot of simulated $P_{f,c}$ values. *Example of an Arrhenius plot of $P_{f,c}$ values, which have been simulated with standard deviation σ_{P_f} = 20%. E_a is derived from the slope of the linear regression (dashed line), which equals $-E_a/R$, according to* ***Eqn. 4****.*

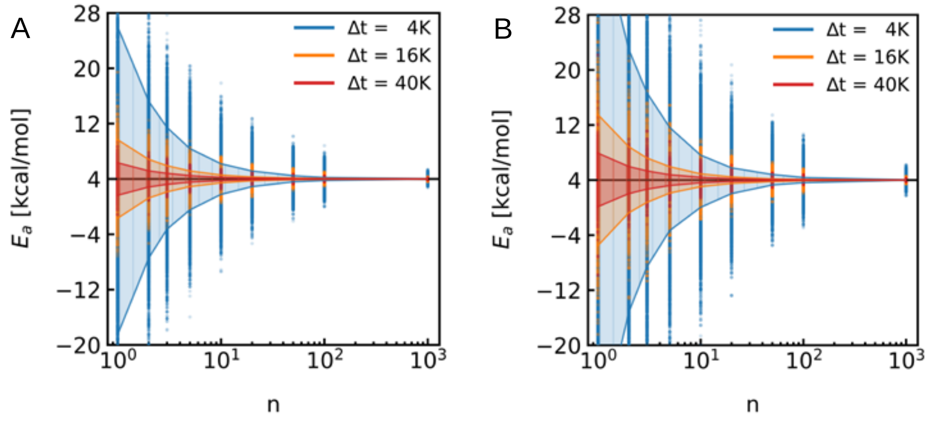

Supplementary Figure 4. Confidence interval of E_a for different σ_{P_f} and varying temperature ranges. *Values of E_a which were calculated from an Arrhenius plot using P_f values and temperature ranges of 4K (blue), 16K (orange) and 40K (red) are represented as dots. The solid lines show the corresponding confidence intervals for the mean E_a's. Plots have been performed for a σ_{P_f} of 20%* ***(A)*** *and 30%* ***(B)****.*

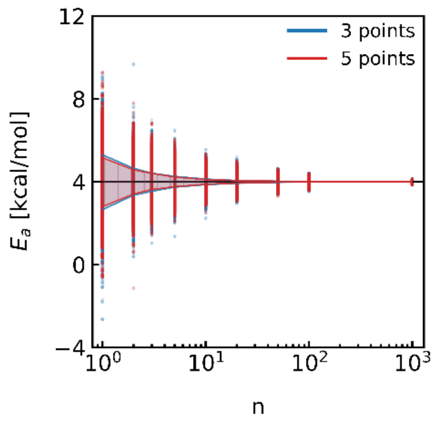

Supplementary Figure 5. Effect of the number of measurement points within a temperature interval on E_a. *Values of E_a which were calculated from an Arrhenius plot using P_f values simulated for 3 temperatures (277K, 297K, 317K) (blue) and 5 temperatures (277K, 287K, 297K, 307K, 37K) (red) are represented as dots. The solid lines show the corresponding confidence intervals for the mean E_a's. Plots have been performed for a σ_{P_f} of 20%.*

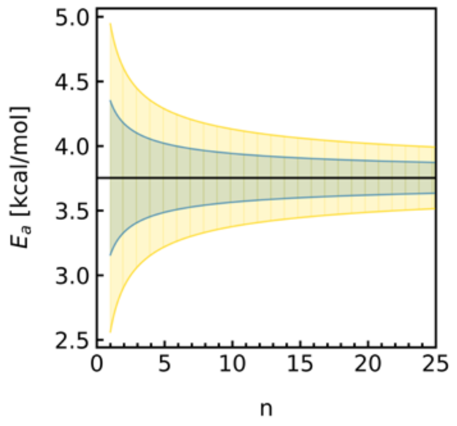

Supplementary Figure 6. Standard error and confidence interval of E_a of AQP1 depending on the number of measurements n. *The standard error of the mean (blue lines) and the confidence interval (yellow lines) show that the true estimate of E_a of AQP1 lies within the borders of the blue and yellow lines with a probability of 68% and 95%, respectively. For the performed measurements the exact values are 3.75 +/- 0.16 kcal/mol with n = 14 measurements.*

Supplementary Note 1. The linearized Eyring equation can be written as:

$$ln\frac{k}{T} = \frac{-\Delta H^{\ddagger}}{R} \cdot \frac{1}{T} + ln\frac{\kappa k_B}{h} + \frac{\Delta S^{\ddagger}}{R} \tag{1}$$

Where k is the rate constant, T the absolute temperature, $\Delta H^{\ddagger}$ the enthalpy of activation, R the gas constant, κ the transmission coefficient, k_B the Boltzmann constant, h the Planck's constant and $\Delta S^{\ddagger}$ the entropy of activation.

The values of $\Delta H^{\ddagger}$ and $\Delta S^{\ddagger}$ can be determined from the rate constants at different temperatures from a $ln\frac{k}{T}$ versus $\frac{1}{T}$ plot. The linear equation has a negative slope $\frac{-\Delta H^{\ddagger}}{R}$ and a y-intercept of $ln\frac{\kappa k_B}{h} + \frac{\Delta S^{\ddagger}}{R}$.

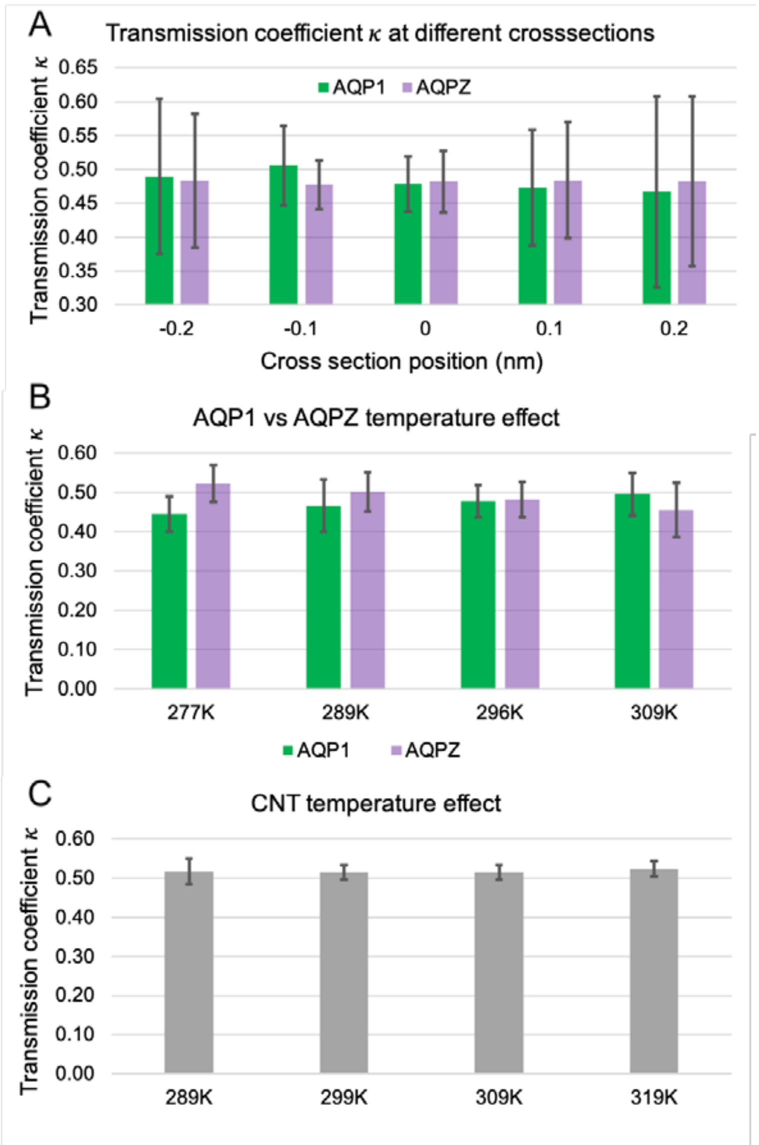

Supplementary Figure 7. Summarized transmission coefficients of AQP1, AQPZ and nCNTPs. (A) *The transmission coefficients κ determined for different cross sections,* z_{div}*, at 296 K similar to* ***Figure 6***. ***(B)*** *Temperature dependence of κ for AQP1 and AQPZ.* ***(C)*** *Temperature dependence of κ for nCNTPs.*

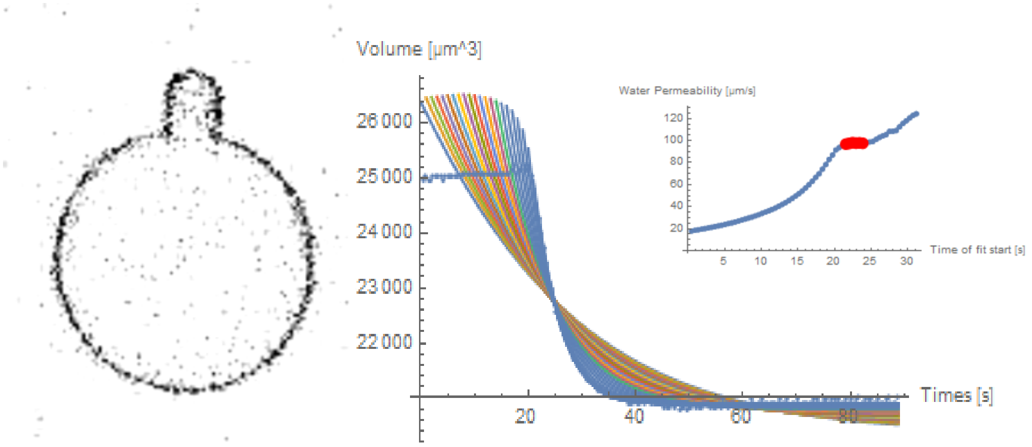

Supplementary Figure 8. Fit of GUV deflation with varying starting points. *Vesicle deflation starts before the whole GUV is exposed to the osmotic gradient. Therefore, time points before and after the visual onset of shrinkage (t = 0) are considered as starting points of evaluation (13). The inverted gray scale laser scanning microscopy videomicrograph was analysed to obtain the time dependent volume change (blue dots). Every frame, where not more than half of the shrinkage has already occurred is used as a starting point of the fitting routine (analytical fits are shown as solid lines). For the determination of* P_f *the region of interest (ROI) for the starting points begins 0.5 s after the visual onset of shrinkage, as this is the maximum time needed for perfect mixing of the osmotic gradient at the GUV surrounding (13) and ends before half of the shrinkage has occurred. The inset shows the determined* P_f *values (blue dots) for each starting point and within the ROI (red dots). The latter are averaged to obtain the final* P_f*.*

Supplementary References

1. A. N. van Hoek, A. S. Verkman, Functional reconstitution of the isolated erythrocyte water channel CHIP28. *J. Biol. Chem.* **267**, 18267-18269 (1992).
2. Z.-J. Yan *et al.*, Artificial Aquaporin That Restores Wound Healing of Impaired Cells. *J. Am. Chem. Soc.* **142**, 15638-15643 (2020).
3. R. H. Tunuguntla *et al.*, Enhanced water permeability and tunable ion selectivity in subnanometer carbon nanotube porins. *Science* **357**, 792-796 (2017).
4. M. L. Zeidel, S. V. Ambudkar, B. L. Smith, P. Agre, Reconstituion of functional water channels in liposomes containing purified red cell chip28 protein. *Biochemistry* **31**, 7436-7440 (1992).
5. L. A. Coury *et al.*, Reconstitution of water channel function of aquaporins 1 and 2 by expression in yeast secretory vesicles. *Am J Physiol* **274**, F34-42 (1998).
6. M. Erbakan *et al.*, Molecular cloning, overexpression and characterization of a novel water channel protein from Rhodobacter sphaeroides. *PLoS One* **9**, e86830 (2014).
7. M. Kumar, M. Grzelakowski, J. Zilles, M. Clark, W. Meier, Highly permeable polymeric membranes based on the incorporation of the functional water channel protein Aquaporin Z. *Proc Natl Acad Sci U S A* **104**, 20719-20724 (2007).
8. M. J. Borgnia, D. Kozono, G. Calamita, P. C. Maloney, P. Agre, Functional reconstitution and characterization of AqpZ, the E. coli water channel protein. *J. Mol. Biol.* **291**, 1169-1179 (1999).
9. W. Xie, J. Wei Jun Low, A. Armugam, K. Jeyaseelan, Y. Wah Tong, Regulation of Aquaporin Z osmotic permeability in ABA tri-block copolymer. *AIMS Biophysics* **2**, 381-397 (2015).
10. P. Pohl, S. M. Saparov, M. J. Borgnia, P. Agre, Highly selective water channel activity measured by voltage clamp: analysis of planar lipid bilayers reconstituted with purified AqpZ. *Proc Natl Acad Sci U S A* **98**, 9624-9629 (2001).
11. M. J. Borgnia, P. Agre, Reconstitution and functional comparison of purified GlpF and AqpZ, the glycerol and water channels from Escherichia coli. *Proc Natl Acad Sci U S A* **98**, 2888-2893 (2001).
12. J. Wachlmayr *et al.*, Scattering versus fluorescence self-quenching: more than a question of faith for the quantification of water flux in large unilamellar vesicles? *Nanoscale Adv* **4**, 58-76 (2021).
13. D. Boytsov, C. Hannesschlaeger, A. Horner, C. Siligan, P. Pohl, Micropipette Aspiration-Based Assessment of Single Channel Water Permeability. *Biotechnol J* **15**, e1900450 (2020).
